# Health Occupation and Job Satisfaction: The Impact of Psychological Capital in the Management of Clinical Psychological Stressors of Healthcare Workers in the COVID-19 Era

**DOI:** 10.3390/ijerph19106134

**Published:** 2022-05-18

**Authors:** Pasquale Caponnetto, Silvia Platania, Marilena Maglia, Martina Morando, Stefania Valeria Gruttadauria, Roberta Auditore, Caterina Ledda, Venerando Rapisarda, Giuseppe Santisi

**Affiliations:** 1 Section Psychology, Department of Educational Sciences, University of Catania, 95124 Catania, Italy; pcapon@unict.it (P.C.); m.maglia@unict.it (M.M.); martina.morando@phd.unict.it (M.M.); stefigrutt@gmail.com (S.V.G.); gsantisi@unict.it (G.S.); 2 Center of Excellence for the Acceleration of Harm Reduction (COEHAR), University of Catania, 95124 Catania, Italy; 3 CTA Psychiatric Rehabilitation and Research, 95030 Mascalucia, Italy; auditore.rob@gmail.com; 4 Department of Clinical and Experimental Medicine, Occupational Medicine, University of Catania, 95123 Catania, Italy; cledda@unict.it (C.L.); vrapisarda@unict.it (V.R.)

**Keywords:** job satisfaction, psychological capital, stress, wellbeing, COVID-19

## Abstract

The COVID-19 pandemic greatly impacted global health. Frontline healthcare workers involved in the response to COVID-19 faced physical and psychological challenges that threatened their wellbeing and job satisfaction. The pandemic crisis, alongside pre-existing critical issues, exposed healthcare workers to constant emotional fatigue, creating an increased workload and vulnerability to stress. Maintaining such stress levels increased their levels of anxiety, irritability and loneliness. Evidence shows that the Psychological Capital (PsyCap) was a strong protective factor against these stressors. The aim of this study was to analyze the level of job satisfaction among health workers facing the COVID-19 pandemic. The possible antecedent factors to satisfaction and the role that PsyCap plays in preserving and fostering higher levels of job satisfaction were investigated. A total of 527 healthcare workers from different areas of Italy were recruited for the study. The results revealed that psychological stress factors have a considerable impact on job satisfaction. All four predictors (Stress Vulnerability, Anxiety Symptoms, Loneliness and Irritability) had the potential to decrease job satisfaction. Loneliness had a more significant effect than other factors assessed in this study. Moreover, the results showed how PsyCap could decrease the effects of psychological stressors on job satisfaction. Consistent with previous studies, our findings show that PsyCap could alleviate negative impacts in work-related circumstances.

## 1. Introduction

The emergency triggered by COVID-19 drastically exacerbated an existing critical structural situation, which further exposed the health system and individual health workers to an unprecedented stress level [1].

Frontline health workers had to immediately adapt to new rhythms, new protocols and new criticalities, and they experienced an increased awareness of being exposed to a serious health risk as they represented a large number of those infected by COVID-19 [2,3,4]. Under conditions such as fatigue, an increased workload and high psychological stress, the perception of the quality of their working life deteriorated [5]. Since the beginning of the pandemic, numerous publications and authors have reported that anxiety, depression and stress are prevalent among frontline healthcare workers [6,7,8,9]. However, a very limited number of studies examine the extent to which and the ways in which the construct of job satisfaction has been affected. Among the published research, however, the results are very clear: healthcare workers around the world are largely dissatisfied with their work situation in the context of the COVID-19 pandemic [10,11,12].

Furthermore, several studies reveal that the physical and psychological symptoms of anxiety and depression lead to poor work performance and significantly increase accident risk [13,14,15,16]. The impact of anxiety on work performance is significant and affects the job both on an individual and organizational level. At the level of the individual employee, it leads to compromised work performance, accidents, and sickness absence. At the organizational level, there are repercussions on productivity, staff morale, accidents, absenteeism and staff turnover [17].

Job satisfaction is defined as an employee’s fulfillment from his/her job, describing a positive emotional response that is achieved as a result of a self-evaluation of their experience at work [18,19]. Job satisfaction is simply an indicator of the extent to which staff enjoy their jobs [19,20]. High job satisfaction is known to have a direct and positive effect on employees’ self-esteem, self-efficacy, morale, performance, and effectiveness, likewise reducing levels of illness, stress, tension, anxiety, complaints, absenteeism, and turnover [12,20]. Analyzing the job satisfaction of healthcare workers is a crucial factor for both healthcare workers and healthcare management. Indeed, it would appear that high levels of satisfaction are directly related to a higher quality of care and service provided, and to greater patient adherence to treatment and higher patient satisfaction [21,22]. This feature is of great importance, not only in emergency conditions such as COVID-19, but generally in terms of practical and managerial implications. Taking care of staff’s Human Resources (HR) and contributions to their mental and physical well-being have a potential direct effect on the quality of care and management of patients. Over time, the literature has differentiated and identified different groups of drivers that precede and predispose health workers’ job satisfaction [23,24,25,26]. Specifically, a group of more traditional drivers, consisting of personal and work-related factors (e.g., age, salary and workload) and a group of drivers more typical of emergency situations, such as that related to COVID-19 (e.g., fear of infecting significant others or risk of exposure to the virus) exist. In this investigation, the antecedent drivers considered were extracted and used after considering this distinction. As traditional drivers, vulnerability to stress and irritability-related symptoms were identified, whereas anxiety-related symptoms and feelings of loneliness were selected as drivers more related to the current pandemic, also by considering the previously mentioned literature.

Other studies on health professionals’ job satisfaction [27,28] revealed a high incidence of PTSD—Post Traumatic Stress Disorder among health professions. Epidemiological data highlight how the organizational, logistical, and operational criticalities present in the structures can produce real traumas, seriously damaging the quality of life of health professionals and, inevitably, that of the service. Moreover, emerging studies indicate that COVID-19 is a traumatic stressor event capable of eliciting Post-traumatic Stress Disorder-related responses and aggravating other related mental health issues (i.e., anxiety, depression, psychosocial functioning, etc.) [29,30].

Worsening working conditions correspond to a greater belief in the opinion that work plays a role in the deterioration of the psychophysical health of respondents [31].

The individual critical issues that constitute work stress can be multiple and should not be underestimated. In the first instance, it turned out that one of the factors of greatest stress is related to communicative confusion, which creates mental overload and physical fatigue. An overall reading suggests that the matrix of these critical issues is of an organizational nature, and that it acts not only directly on specific tasks—making them more tiring—but also on the perception of oneself and one’s placement in the workflow [32]. Chaotic communication and planning create frustration, a sense of injustice and a lack of recognition of one’s own value. The pandemic crisis, in addition to the pre-existing criticalities, has placed healthcare personnel in constant emotional fatigue, creating greater amounts of stress. The progression of the infection and the maintenance of these stress levels for a long time has increased the chances of developing greater levels of anxiety, irritability and loneliness [33]. A condition of stress that, when predicting work burn-out events, undermines work performance and, consequently, care outcomes [34]. Despite the difficulties encountered by health care workers, not all of them are destined to suffer the effects of work-related stress, or experience job burnout or severe dissatisfaction. Much scientific evidence has in fact confirmed that it is possible to counteract this fatigue through a decisive protective factor that constitutes the general well-being of the worker and influences their performance, i.e., the Psychological Capital (PsyCap).

When conducting our study, we found that there was limited literature on the issue of health professionals’ satisfaction during the emergency phase in Italy. The organization aims to realize valuable approaches to mitigate the risk of infection in the workplace that could improve job satisfaction and well-being [35,36,37].

Therefore, the aim of our study was to evaluate the levels of job satisfaction among health professionals, investigating both the specific antecedent factors (more traditional and more related to the specific situation) and the impact that psychological capital plays in preserving and fostering higher levels of job satisfaction. The present study will therefore be used to carry out targeted health-promotion interventions among healthcare workers.

## 2. Predictors of Job Satisfaction

The health workers who protect and improve the health of individuals are crucial to the success of health systems, and the achievement of health goals is dependent on their work and commitment, both at a national and global level. This was especially true during the COVID-19 pandemic, where these workers worked in extreme, risky situations while maintaining professionalism and devotion. However, in order to effectively respond to the health needs of populations, health workers themselves must show good health, otherwise they will suffer direct and indirect negative effects. Indeed, while in traditional, pre-pandemic times, health workers were faced with various psychosocial pressures, during the pandemic these challenges certainly increased.

Working night shifts, long working hours, responding quickly to patients’ requests for care, medical disputes, enduring violence in the workplace, and emotional distress due to poor interactions with patients and colleagues, and poor promotion prospects are just some of the daily problems that medical staff have always faced, and which during the pandemic placed an even greater strain on such workers. Constant exposure to these psychosocial risks has a negative impact on the health of healthcare workers and the care service provided.

Therefore, healthcare professionals seem to be the most vulnerable to stress among all occupational groups due to the nature of their work environment [38].

For these reasons, the following hypotheses were proposed:

**H1.**  *The vulnerability to stress is negatively related to job satisfaction.*

**H2.**  *The anxiety symptoms is negatively related to job satisfaction.*

Recently published studies [39] consider that employees’ well-being plays an important role in job satisfaction and subsequent retention, especially when employees perceive positive emotions at work. Indeed, job satisfaction leads to an immediate return for the organisation, both at an individual, group and organisational level, by influencing these areas on an emotional and cognitive level [40]. Job satisfaction is believed to be a good predictor of absenteeism [41] and turnover intention [42] and specifically in the health sector, the literature has widely shown that a high job satisfaction of physicians benefits their physical and mental health [43]. Indeed, it may be a protective factor against burnout, intention to leave, absenteeism [41], and turnover intention [44,45].

One element that is significantly linked to the pandemic situation is loneliness. Loneliness occurs when people experience a sense of loneliness and when, regardless of the number of contacts, they begin to lack meaningful social relationships. The current epidemic situation and the relative need to maintain social distance has worsened interpersonal relationships of all kinds, thus limiting social support, which may also contribute to the growing feeling of loneliness [46].

The problem of the presence of loneliness in the professional life of medical employees is not described in detail in the literature. In recent years, studies have been published on the existence of loneliness, especially among physicians, where the authors state that loneliness is common in the work of physicians and is associated with burnout and low job satisfaction [47,48]. In a study involving 401 family doctors, the incidence of loneliness was 44.9%. An analysis of the results showed that physicians who experienced a greater sense of loneliness more often reported at least one of the symptoms of professional burnout. In other studies, loneliness has been identified as one of the main stressors in the work of nurse managers [49]. For this reason the following was hypothesized:

**H3.**  *The loneliness is negatively related to job satisfaction.*

Finally, in the examination of possible predictors of job satisfaction most related to the pandemic, irritability and anger were highlighted. The unexpected and difficult conditions into which COVID-19 forced health workers generated feelings of fear and helplessness in health workers, heavily influencing their mental health conditions and causing feelings of irritability, frustration and anger. In addition, an increased workload and job deterioration may also have exacerbated the anger state of health care workers [50]. Based on these observations, it seemed interesting to investigate the following within the scope of the present study:

**H4.**  *The irritability is negatively related to job satisfaction.*

## 3. Psychological Capital as a Protective Factor

The literature review revealed that health care workers are at a significantly higher risk not only of harmful physical effects from COVID-19, but also of harmful psychological sequelae. However, in addition to the negative predictors that significantly influence the job satisfaction and quality of life of such workers, important protective factors can also be identified. The presence of children, a strong social and family network, team cohesion and shared responsibility among colleagues, adequate personal protective equipment, the use of humor and planning as coping strategies, and the ability to talk to someone about one’s experiences seem to be protective factors for the mental health of health care workers, as reported in some Italian studies [51,52]. In our study, therefore, we expressed interesting in studying the role of Psychological Capital, specifically as a mediator in the relationship between the previously mentioned predictors and job satisfaction.

Psychological Capital is a combination of several psychological variables, such as self-efficacy, optimism, hope, and resilience, and it has been defined as “an individual’s positive psychological states”. The PsyCap is characterised by the following: “(1) having self- confidence, self-efficacy; (2) making a positive attribution, optimism; (3) being able to redirect paths towards goals hope; and (4) being able to sustain, overcome and bounce back from problems and adversity, resilience” [53] (p. 3). Empirical evidence shows that improving workers’ overall PsyCap levels had an effect in reducing stress symptoms, turnover rates and generally led to workers experiencing a better quality of life and improved mental and physical well-being [54,55,56]. In the specific case of this study, the PsyCap as an aggregate factor was interpreted and used as a resource and protective factor in raising values of job satisfaction [57,58,59]. It was then assumed that:

**H5.**  *The Psychological Capital is positively related to job satisfaction.*

**H6.**  *The vulnerability to stress is negatively related to Psychological Capital.*

**H7.**  *The anxiety symptoms is negatively related to Psychological Capital.*

**H8.**  *The loneliness is negatively related to job Psychological Capital.*

**H9.**  *The irritability is negatively related to job Psychological Capital.*

**H10.**  *The Psychological Capital (PsyCap) mediated the relationship between the drivers before mentioned and the job satisfaction.*

**H10a.**  *Vulnerability to stress will have an indirect effect on job satisfaction through the role of Psychological Capital.*

**H10b.**  *Anxiety Symptoms will have an indirect effect on job satisfaction through the role of Psychological Capital.*

**H10c.**  *Loneliness will have an indirect effect on job satisfaction through the role of Psychological Capital.*

**H10d.**  *Irritability will have an indirect effect on job satisfaction through the role of Psychological Capital.*

## 4. Method

### 4.1. Participants and Procedure

The convenience sampling technique was used in this study, which is a method where the selection of participants is based on their ready availability [60] and we enrolled participants from the health workers population. Data were collected from May 2020 to November 2021, using an online survey involving several Italian hospitals. The HR (Human Resource) departments contributed to the study, granting approval for the study and encouraging the distribution of the questionnaire to healthcare workers. Through the hospital’s social media and workgroups, via written correspondence (e.g., email or invitation by letter to participate), the study sample was achieved.

A link, an information sheet, instructions and an informed consent form were presented to each of the respondents. An individual, anonymous and structured questionnaire was used with several standardized and validated scales, that required approximately 20 min to complete. Participation was voluntary, and no incentives to participate were provided. The study was carried out in accordance with the Declaration of Helsinki, and the protocol was authorized by the Internal Ethics Review Board of the Department of Educational Sciences (Section of Psychology) of the University of Catania (Ierb-Edunict-2020/4); all the research procedures followed all the indications provided by the guidelines of the AIP (Italian Association of Psychology) and its Ethical Council.

Five-hundreds and twenty-seven health care workers on the front line of the battle against COVID-19 during the pandemic were enrolled in this study (physicians 35.2%, nurses 48.1%, health care assistants 16.7%; females were 71.7% of the total sample). Participants were from different areas in Italy (North, 36%; Central, 23%; South, 41%). Their age ranged between 24 and 59 (Mage = 36.6, SD = 15.4). As for the educational level, 69.7% had completed a minimum of 17 years of school. Research participants had an average seniority of 13.5 (SD = 4.3).

### 4.2. Measures

#### 4.2.1. Stress Vulnerability Scale (SVS)

Sensitivity to psychological stress was investigated using the Stress Vulnerability Scale (SVS) devised by Miller and Smith in 1985 [61]. This scale is made up of 20 items. (e.g., *I get seven to eight hours of sleep at least four nights a week; I am in good health, including eyesight, hearing, dental health, etc.)*. Each individual’s ability to withstand physical and psychological stress was evaluated. Each test item evaluated the temporal frequency with which each statement was true for the subject examined. Each response was measured on a Likert scale from 1 (always) to 5 (never).

#### 4.2.2. Anxiety Symptoms

The assessment of the presence and severity of anxiety symptoms was investigated with the GAD-7 (General Anxiety Disorder) scale in both the English and French versions [62,63]. The self-assessment test consists of seven items that investigate the presence or absence of anxiety symptoms and intensity in the last 2 weeks, e.g., *Over the last two weeks, how often have you been bothered by the following problems? Feeling afraid, as if something awful might happen; Worrying too much about different things*). Answers were provided on a scale from 0 to 3, where 0 indicated the absence of symptom “For Nothing”, 1 indicated the presence for “Several days”, 2 indicated a higher frequency for “More than half the days” and 3 indicated the maximum frequency “Most days”. A cut-off score ≥10 out of a total of 21 indicated the presence of moderate to severe anxiety disorder. The higher the score, the greater the severity of the anxiety disorder. The GAD-7 scale was developed with the clear objective of screening patients with generalised anxiety disorder. The scale has also been widely used in clinical and research settings to monitor the severity of GAD symptoms. It has proven to be a reliable and valid instrument, and its seven items reflect some of the diagnostic domains of GAD in the Diagnostic and Statistical Manual of Mental Disorders, Fifth Edition (DSM-V). GAD is highly comorbid with other anxiety disorders and typically precedes the occurrence of comorbidities, which has contributed to the conceptualisation of GAD as a ‘core’ anxiety disorder [64,65]. Using this threshold, the GAD-7 has a sensitivity of 89% and specificity of 82% for generalized anxiety disorder [66,67].

#### 4.2.3. Loneliness

The UCLA Loneliness Scale [68,69] was used to investigate the construct of loneliness. The test examines the presence of loneliness and the frequency with which the subject perceives it. To each question, the subject could answer on a scale from 1 to 3 (1 = “Almost never”; 2 = “Sometimes”; 3 = “Very often”), the presence of high scores in the three items (i.e., “*How often have you felt a lack of companion-ship*”, “*How often have you felt left out*”, “*How often have you felt isolated from others*”) revealed a higher frequency of the feeling of loneliness among the interviewees.

#### 4.2.4. Irritability

For the assessment of irritability symptoms, a brief self-report measure assessing the level of frustration and irritability experienced by the participants in the last two weeks was used. We adopted the Brief Irritability Test scale, in its English and French versions [70] (BITe). Consisting of just five items, interviewees responded on a 6-point frequency scale, from 1 = Never to 6 = Always (e.g., *Other people have been getting on my nerves; Things have been bothering me more than they normally do*). The BITe has been validated in both healthy and patient samples and has demonstrated adequate evidence of validity and reliability [70].

#### 4.2.5. Compound PsyCap Scale (CPC-12)

The Compound Psychological Capital Scale (CPC-12) was used for the assessment of the psychological capital construct, proposed by Lorenz et al. [71], the Italian version of which was constructed by Platania and Paolillo [72]. The construct of Psychological Capital draws from positive psychology and more specifically from positive organisational behaviour. It extends the traditional binomial of human and social capital by representing the state of positive psychological development of an individual that is characterised by the following four elements: self-efficacy, optimism, hope and resilience. The scale consists of 12 items, containing the four components of psychological capital [*Hope (measured by three items, e.g., “Right now, I see myself as being pretty successful”), Resilience (measured by three items, e.g., “When I’m in a difficult situation, I can usually find my way out of it”), Optimism (measured by three items, e.g., “Overall, I expect more good things to happen to me than bad.”) and Self-Efficacy (measured by three items, e.g., “I can solve most problems if I invest the necessary effort”)*]. For each statement, answers were provided through a 7-point Likert scale from 1 = strongly disagree to 7 = strongly agree.

#### 4.2.6. Job Satisfaction Survey (JSS)

The construct of job satisfaction was examined with the Job Satisfaction Scale, the Italian version for which was constructed by Platania et al. [73]. The scale, composed of 36 items, measures the construct from a multidimensional perspective, taking into account the following nine aspects of organisational life: Pay, Promotion, Supervision, Fringe Benefits, Contingent Rewards, Operating Conditions, Employees, Nature of Work and Communication (e.g., *My efforts to do a good job are seldom blocked by red tape; I find I have to work harder at my job because of the incompetence of people I work with*). Each participant responded to the items by indicating their level of agreement on a 6-point Likert scale, from 1 to 6. The total score on this scale ranged from 36 to 216. Satisfaction and dissatisfaction scores could be calculated in aggregate or separated by factors. For each of the nine factors, scores from 4 to 12 represent dissatisfaction, from 16 to 24 show satisfaction, and those between 12 and 16 represent ambivalence. For the total number of items, the ranges were 36 to 108 for dissatisfaction, 144 to 216 for satisfaction, and 108 to 144 for ambivalence.

### 4.3. Data Analysis

In order to perform a descriptive and correlational analysis of the variables in this study, the statistical package SPSS (version 27.0 for Windows; IBM Corp., Armonk, NY, USA) was used. By contrast, structural equation models (SEM), completed in AMOS 27.0, were used to test the hypothesised model [74].

Cronbach’s alpha was calculated in order to ensure the internal consistency and reliability of all the scales used in this study [75]. This important procedure demonstrated the value of the scales used, showing how they can be both credible and reproducible, even in different contexts.

Convergent validity and discriminant validity were also calculated and evaluated to confirm good content validity. Convergent validity was confirmed by the factor loading size, average variance extracted (AVE) and composite reliability (CR) values. CR should be greater than the AVE and the AVE should be greater than 0.5 to confirm convergent validity [76].

The square root of the AVE values was also included and compared with the correlation coefficients between the constructs. Discriminant validity is achieved if these values (AVE square roots) are greater than the correlations between two variables [76].

A set of confirmatory factor analyses (CFA) were run on the dataset to identify the model that best fit the dataset. Harman’s single-factor test [77] was calculated to examine the common variance problem (CMV). The goodness of fit of the model was assessed by means of several indexes. The comparative fit index (CFI), goodness-of-fit statistics (GFI), standardised root mean square residual (SRMR), root mean square error of approximation (RMSEA) and Tucker–Lewis index (TLI) were used. An omnibus cut-off point of 0.90 for the GFI index is traditionally recommended [78]. RMSEA values in the range of 0.05 to 0.10 were considered an indication of discrete adaptation and values above 0.10 indicated marginal adaptation [79]. Therefore, an RMSEA value of between the values of 0.08 and 0.10 was considered to provide a poor fit and a value of below 0.08 was considered to denote a good fit [79]. However, more recently, a cut-off value of close to 0.06 [80] or a strict upper limit of 0.07 [81] appear to be the general consensus. Values for CFI range from 0 to 1, and Bentler and Bonnet [82] recommend that values of above 0.90 indicate a good fit. More recent suggestions asserted that the cut-off criterion should be TLI ≥ 0.95 [80]. For CFI values, a cut-off criterion of CFI ≥ 0.90 was initially proposed, however, other studies have shown that a CFI value of ≥ 0.95 is currently recognised as indicating good adaptation [80,83]. Finally, values for SRMR range from zero to 1.0, with well-fitted models obtaining values of less than 0.05 [84,85]. An SRMR of 0 signifies a perfect fit.

In addition, the χ^2^ and Δχ^2^ values between competing models were also presented as sensitive to sample size [86], and the Akaike information criterion (AIC) and Bayesian information criterion (BIC) were used concomitantly (lower values indicate a better fit). According to the method of Hayes and Preacher, a statistical mediation analysis was conducted [87].

In order to test the indirect effects of the relationship of Psychological Capital, a mediation analysis was performed using a structural equation model. Following the guidance provided by James and colleagues [88] and Shrout and Bolger [89] on expected proximal and distal effects, two regression models were applied simultaneously, assuming that the total effect of the dependent variable on the independent variable differed from the direct effect of the variable. The indirect effect was tested using a bootstrap estimation approach on 2000 samples and a percentile method which corrected for 95% bias [87].

## 5. Results

### 5.1. Descriptive Statistics and Multivariate Normality

As a first step, the distribution of our sample was checked. In Structural Equation Models (SEM), it is very important to check whether the distribution is multivariate as it will determine which estimation method will be used and to what extent the estimates obtained by the most common methods are trustworthy [90]. In Table 1, all the variables that make up the scales of both the first order and second order were used to verify the normality of the distribution. If the distribution was found to be multivariate, each observed variable would have a minimum value, a maximum value, a skewness value and a kurtosis value. Critical values that exceeded +2.00 or that were smaller than −2.00, indicated statistically significant degrees of non-normality. The descriptive statistics in Table 1 show that the data were normally distributed, with acceptable skewness and kurtosis values.

### 5.2. Descriptive Statistic, Correlation, and Reliability

The descriptive statistics and correlation matrix for all variables of the study are reported and described in Table 2. The results revealed that Composite Psychological Capital correlated significantly and positively with Job Satisfaction (r = 0.74, *p* < 0.001), while it negatively correlated with Loneliness (r = −0.75, *p* < 0.001), Irritability (r = −0.56, *p* < 0.001), Anxiety Symptoms (r = −0.28, *p* < 0.001), and Stress Vulnerability (r = −0.35, *p* < 0.001).

On the other hand, the composite reliability and mean variance extracted had of the following values: CR 0.84, AVE 0.71 for stress vulnerability; CR 0.86 and AVE 0.72, for Anxiety Symptoms; CR 0.83 and AVE 0.69, for Loneliness; CR 0.93 and AVE 0.76, for Irritability; CR 0.91 and AVE 0.77, for Compound Psychological Capital (CPC-12); and CR 0.90 and AVE 0.73 for Job Satisfaction. Cronbach’s alpha (α) values were very good, along with CR and AVE, indicating very good overall internal consistency of the scale [91].

### 5.3. CFA to Test the Model

In order to control for the common bias effect that can occur when variables are measured from the same source, a Confirmatory Factor Analysis (CFA) was conducted according to Harman’s one-factor test [92]. The comparison between the hypothesized model and a single-factor model (with all items loading on a single factor) revealed that the former provided a better fit to the data in all CFA fit measures (Model 1, 6-factor model: χ^2^ (166) = 642.05, *p* < 0.001, CFI = 0.89, GFI = 0.92, SRMR = 0.04, RMSEA = 0.065, and AIC = 514.16; Model 2, 1-factor model: χ^2^ (191) = 1917.10, *p* < 0.001, CFI = 0.54, GFI = 0.65, SRMR = not estimable, RMSEA = 0.19, and AIC = 3587.11). The difference between the chi-square models and degrees of freedom was significant Δχ^2^ (25) = 1274.95 (*p* < 0.001). Based on these results, we found no evidence of a common method bias in the data.

### 5.4. Structural Model

**Figure 1:**
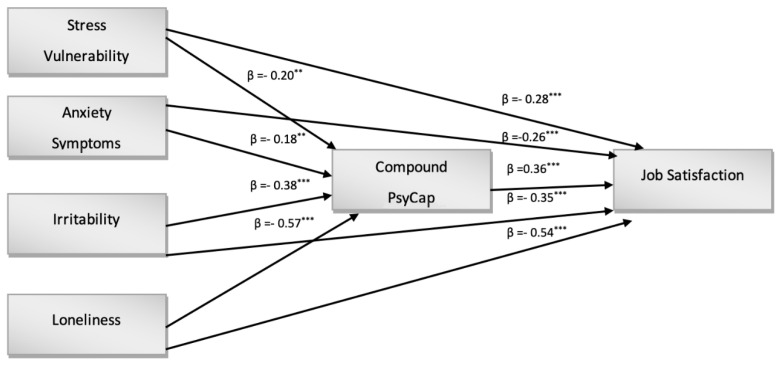
Structural model *** *p* < 0.001, ** *p* < 0.01.

As a direct result of the CFA outcomes, a path diagram based on model 1 was constructed. As shown in Figure 1, a model was maintained that verified the direct impact of Stress Vulnerability, Anxiety Symptoms, Irritability and Loneliness on Psychological Capital and on Job Satisfaction as a second-order variable. The results showed that there was a direct effect of all predictors on Job Satisfaction. In particular, there was a directed effect of Stress Vulnerability (H1, β = −0.28; *p* < 0.001), Anxiety Symptoms (H2, β = −0.26; *p* < 0.001), Irritability (H3, β = −0.35; *p* < 0.001) and Loneliness (H4, β = −0.54; *p* < 0.001). In our study, therefore, all the hypotheses relating to the negative impact that conditions of anxiety, stress, loneliness, frustration, and moodiness can have on the effects related to job satisfaction were confirmed (Figure 1). The same predictors also revealed a direct effect on Compound PsyCap. In detail, there were direct effects of Stress Vulnerability (H6, β = −0.20, *p* < 0.01), Anxiety Symptoms (H7, β = −0.18, *p* < 0.001), Irritability (H8, β = −0.38, *p* < 0.001), and Loneliness (H9, β = −0.57, *p* < 0.001) on Compound PsyCap.

In order to test another objective in the present study (Hypothesis 5), the mediating effects of psychological capital in the relationship between negative predictor effects and job satisfaction were investigated. The results shown in Table 3 correspond to the expected findings. For Stress Vulnerability, Anxiety Symptoms, Irritability and Loneliness, the results confirmed a partial mediation of Compound Psychological Capital in relation to Job Satisfaction; in particular, indirect effects were significant for Stress Vulnerability (H10a, β = 0.06, *p* < 0.001, SE = 0.053, 95% CI = −0.198–−0.079), Anxiety Symptoms (H10b, β = 0.06, *p* < 0.001, SE = 0.043, 95% CI = 0.268–0.513), Irritability (H10d, β = 0. 05, *p* < 0.001, SE = 0.041, 95% CI = 0.192–0.308) and Loneliness (H10c, β = 0.06, *p* < 0.001, SE = 0.057, 95% CI = −0.210–−0.124) [91,92].

## 6. Discussion

In the present study, the main aim was to investigate the ‘health status’ of healthcare workers under conditions of high stress during the pandemic. The healthcare workers most affected were those working on the front line, and therefore, were faced with an unprecedented situation.

In particular, the sudden emergency conditions highlighted the adaptability and coping skills of healthcare workers who were prompted to draw on their own resources in terms of self-efficacy, ability to achieve the required goals, optimism and, above all, resilience in facing the health emergency.

Therefore, it is of utmost importance to determine the psychological actions directed towards the mental health consequences of the global COVID-19 pandemic, as well as the mediating effect that psychological capital could have in the relationship between psychological distress and job satisfaction.

In our study, it emerged that psychological stress-related factors had a considerable impact on job satisfaction. All four of the predictors (Stress Vulnerability, Anxiety Symptoms, Loneliness and Irritability) examined were found to particularly decrease job satisfaction.

Amongst other things, it is interesting to note the more pronounced result of Loneliness, whose impact on job satisfaction was significantly greater than the other factors analyzed.

The aspect of Loneliness in health professionals was also investigated in other studies [93,94] and seems to be a particularly important finding for studies concerned with professionals who work in the environment of a health crisis.

Another objective of our study was to test the indirect effect of psychological capital, as a personal resource to be drawn on in response to the psychological stress arising from the situation. Our study shows how Psychological Capital could diminish the effects of psychological stressors on job satisfaction. Our results are consistent with previous studies that demonstrated that Psychological Capital could alleviate the role in work-related circumstances [72,95].

## 7. Conclusions

Previous studies found that workers with high levels of psychological capital tend to be more confident in mobilizing cognitive resources.

Other studies determined that for health professionals, the higher the psychological capital, the more better adapted they are to showing resilience in uncertain situations and of pursuing pathways to success [96,97].

In addition, recent studies have highlighted the importance of protective factors such as coping and resilience strategies as a response to the stress of health care workers during the pandemic by comparing the first and second waves. The results showed that stress significantly reduced in the second wave when the emergency was better contained, thus revealing that resilience is a fundamental protection factor for the health of workers and that intervention protocols in this sense are necessary [98].

Furthermore, our result regarding a positive correlation between age and psychological capital is in agreement with that of Bozda and Ergün [99], who found a positive relationship between psychological resilience and age, showing that older health workers are better able to cope with crises because they have more extensive experience and a greater skillset.

However, frontline health workers who were less optimistic, motivated and resilient at work (thus having low psychological capital), were more likely to experience greater burnout in the face of anxiety.

Our findings are in line with this research, i.e., hope, optimism, resilience and self-efficacy could alleviate the ways in which employees experience severe psychological stress.

In conclusion, our study suggests, using empirical evidence, that psychological capital is a fundamental variable for improving the effects of stress and anxiety on job satisfaction.

## 8. Limits and Practical Implications

Our aim was to investigate the mediating role of Psychological Capital in the relationship between psychological stress and job satisfaction; further research could help to better understand the ways in which stress vulnerability, anxiety symptoms, irritability and loneliness could affect job satisfaction.

There are several limitations in the present study. First, because it was designed as a study cross-section, we did not establish the relationship between variables. Further input could be obtained through future longitudinal research which could lead to a better understanding of causality among the variables.

Second, the study variables were measured using a self-reported questionnaire, which could impact the results because of the common method of polarization variance.

Furthermore, future research could expand on this topic and investigate the mediating role of Psychological Capital on other multilevel outcome variables to further verify the personal and organizational implications of health workers.

Another limitation of our research concerns external validity, in that our results are not comparable with those of other countries that have experienced the same problem and therefore cannot be generalized. External validity captures the extent to which inferences drawn from a given study’s sample apply to a broader population or other target populations, and therefore represents a limitation in this study.

Regarding the practical implications that may derive from this study, the results suggest how, in a pandemic situation, involving frontline health workers, the effect of psychological capital could actually reduce the psychological stress arising from the work situation experienced and this implies that job satisfaction is not diminished.

As confirmed by other research that highlighted the role of psychological capital in the same contexts and in the same situation, psychological capital is the most suitable personal resource that could be developed through training and coaching programs [99,100,101,102,103].

It is therefore necessary to develop psychological interventions that assist healthcare personnel in acquiring important resources such as resilience, optimism, self-efficacy, etc., that are useful for facing moments of crisis such as the COVID-19 pandemic.

For example, it could be valid to develop and adapt a mental health support plan as it has already been done in other countries, within which various intervention measures have been established, including measures that encourage workers in first line response capacities to make use of free online psychological first aid training, both to improve their awareness of potential mental health risks, and to improve their understanding of when to refer people to specialist services [100,101].

In conclusion, we believe that our research contributed to recent efforts to uncover the mediating role of psychological capital in the relationship between psychological stress and job satisfaction among frontline health professionals since the outbreak of the global COVID-19 epidemic.

In addition, these findings encourage the development of human resource management practices such as programs that aim to increase the performance and health of the psychological capital of frontline health workers.

## Figures and Tables

**Table 1:** Descriptive statistics (range [minimum/maximum], mean [M], including standard error [SE], standard deviation [SD], skewness and Kurtosis).

	N	Min	Max	M	SE	SD	Skewness	Kurtosis
Stress Vulnerability	527	1.40	4.80	4.22	0.045	1.06	−0.361	−0.812
Anxiety Symptoms	527	1.00	2.70	2.11	0.041	0.89	−0.791	1.541
Loneliness	527	1.00	3.00	2.47	0.072	0.95	−0.236	−0.454
CPC-12 Hope	527	2.00	6.00	5.88	0.028	−0.89	−0.891	1.321
CPC-12 Optimism	527	1.00	6.00	5.11	0.061	1.03	−0.896	1.214
CPC-12 Resilience	527	2.00	6.00	5.65	0.057	1.17	0.975	1.521
CPC-12 Self Efficacy	527	1.00	6.00	5.31	0.068	1.12	−0.852	1.340
JSS Pay	527	5.00	19.00	12.91	0.106	2.14	−0.85	0.256
JSS Promotion	527	4.00	20.00	10.92	0.125	2.83	0.217	−0.37
JSS Supervision	527	6.00	19.00	11.62	0.085	1.86	0.085	0.497
JSS Fringe_Benefits	527	4.00	20.00	12.52	0.054	2.02	0.095	0.849
JSS Contingent rewards	527	6.00	20.00	13.11	0.073	2.78	−0.192	−0.752
JSS Operating procedures	527	4.00	20.00	12.67	0.101	3.06	−0.209	−0.307
JSS Co-workers	527	6.00	20.00	13.65	0.104	2.25	−0.116	0.420
JSS Nature_of_work	527	8.00	20.00	14.33	0.092	2.24	−0.561	0.054
JSS Communication	527	5.00	20.00	10.79	0.083	2.62	0.263	−0.241
Irritability	527	1.00	6.00	4.68	0.048	1.10	0.289	0.874

**Table 2:** Descriptive statistic, correlation, and reliability.

	M	SD	α	1	2	3	4	5	6	7	8
1. Stress Vulnerability	4.22	1.06	0.87	-							
2. Anxiety Symptoms	2.11	0.89	0.89	0.57 **	-						
3. Loneliness	2.47	0.95	0.85	0.48 **	0.29 **	-					
4. CPC-12	5.96	1.15	0.92	−0.35 **	−0.28 **	−0.75 **	-				
5. JSS	5.31	1.08	0.91	−0.37 **	−0.32 **	−0.67 **	0.74 **	-			
6. Irritability	4.68	1.10	0.92	0.38 **	0.46 **	0.51 **	−0.56 **	−0.54 **	-		
7. Age	36.6	15.4	-	−0.19 *	−0.16 *	−0.21 **	0.27 *	−0.15 *	−0.17 *	-	
8. Gender	-	-	-	0.24 **	0.25 **	0.29 **	0.20 **	0.23 **	0.31 **	−0.23 **	-

Note: CPC-12 = Compound Psychological Capital; JSS = Job Satisfaction Survey; *p* scores: * <0.05, ** <0.001.

**Table 3:** Standardized indirect effects from Stress Vulnerability, Anxiety Symptoms, Irritability and Loneliness to Job satisfaction through Compound PsyCap (*** *p* < 0.001, ** *p* < 0.01).

Predictor		Mediator		Outcome	β	SE	BC 95% CI	
							LL	UL
Stress Vulnerability	→	Compound PsyCap	→	Job Satisfaction	0.06 ***	0.05	−0.198	−0.079
Anxiety Symptoms	→	Compound PsyCap	→	Job Satisfaction	0.06 ***	0.04	0.269	0.513
Irritability	→	Compound PsyCap	→	Job Satisfaction	0.05 **	0.04	0.192	0.308
Loneliness	→	Compound PsyCap	→	Job Satisfaction	0.06 ***	0.06	−0.210	−0.124

Legend: BC = Booustrap confidence; CI = Confidence Interval, LL = Lower Level; UL = Upper Level.
